# Early-life stress induces EAAC1 expression reduction and attention-deficit and depressive behaviors in adolescent rats

**DOI:** 10.1038/s41420-020-00308-9

**Published:** 2020-08-08

**Authors:** Han-Byeol Kim, Ji-Young Yoo, Seung-Yeon Yoo, Sang Won Suh, Seoul Lee, Ji Hye Park, Jun-Ho Lee, Tai-Kyoung Baik, Hye-Sun Kim, Ran-Sook Woo

**Affiliations:** 1 Department of Anatomy and Neuroscience, College of Medicine, Eulji University, Daejeon, 34824 Republic of Korea; 2 Department of Physiology, College of Medicine, Hallym University, Chuncheon, 24252 Republic of Korea; 3 Department of Pharmacology and Brain Research Institute, College of Medicine, Wonkwang University, Jeonbuk, 54538 Republic of Korea; 4 Department of Emergency Medical Technology, Daejeon University, Daejeon, 34520 Republic of Korea; 5 Department of Pharmacology, College of Medicine, Seoul National University, Seoul, 110-799 Korea; 6 Seoul National University College of Medicine, Bundang Hospital, Sungnam, 13620 Republic of Korea

**Keywords:** Depression, Molecular neuroscience

## Abstract

Neonatal maternal separation (NMS), as an early-life stress (ELS), is a risk factor to develop emotional disorders. However, the exact mechanisms remain to be defined. In the present study, we investigated the mechanisms involved in developing emotional disorders caused by NMS. First, we confirmed that NMS provoked impulsive behavior, orienting and nonselective attention-deficit, abnormal grooming, and depressive-like behaviors in adolescence. Excitatory amino acid carrier 1 (EAAC1) is an excitatory amino acid transporter expressed specifically by neurons and is the route for the neuronal uptake of glutamate/aspartate/cysteine. Compared with that in the normal control group, EAAC1 expression was remarkably reduced in the ventral hippocampus and cerebral cortex in the NMS group. Additionally, EAAC1 expression was reduced in parvalbumin-positive hippocampal GABAergic neurons in the NMS group. We also found that EAAC1-knockout (EAAC1−/−) mice exhibited impulsive-like, nonselective attention-deficit, and depressive-like behaviors compared with WT mice in adolescence, characteristics similar to those of the NMS behavior phenotype. Taken together, our results revealed that ELS induced a reduction in EAAC1 expression, suggesting that reduced EAAC1 expression is involved in the pathophysiology of attention-deficit and depressive behaviors in adolescence caused by NMS.

## Introduction

Early-life stressful events have detrimental effects on the brain and are risk factors for behaviors that are associated with the etiology of several psychiatric disorders. Childhood adversities are associated with maladaptive family functioning that has been related to one-third of adult psychopathology^1^. Elucidating the mechanism of the correlation between early-life manipulations and subsequent disease is difficult to be proven in humans^2^. In animal models, early developmental manipulations involving maternal care has been identified as an ethologically relevant stressor that induces cognitive and emotional dysfunction throughout life^3^. Neonatal maternal separation (NMS) enhances responses to aversive and appetitive stimuli more cautiously toward fear signals in the environment^4,5^. The postnatal period is accompanied by significant maturation of neuronal systems. Experiencing stressful events results in increased neurochemical, neurobehavioral, and immune-inflammatory abnormalities in subsequent years^6^.

Glutamate is the primary excitatory neurotransmitter in the central nervous system and plays a central role in the neurotransmission of ~80% of synapses^6,7^. Dysregulation of glutamatergic neurotransmission is related to stress- and depressive-like behaviors^8,9^. Excitatory amino acid carrier 1 (EAAC1, also referred to as EAAT3) is one subtype of the excitatory amino acid transporter (EAAT) family^10^. Compared with other subtypes, including the glutamate aspartate transporter (GLAST, also referred to as EAAT1), glutamate transporter-1 (GLT1, also referred to as EAAT2), EAAT4 and EAAT5, EAAC1 plays a more significant role in cysteine transport in the brain^11^. EAAC1 was first described as a neuronal glutamate transporter^10^, although it has now been shown to play only a minor role in glutamate removal from the extracellular space, as this task is primarily performed by astrocyte glutamate transporters such as GLT1 and GLAST^12,13^. EAAC1 is expressed at presynaptic GABAergic terminals, where the uptake of glutamate could contribute to GABA synthesis^14–16^. The loss of brain EAAC1 expression interferes with GABA synthesis and results in epilepsy^17,18^. EAAC1 expression is altered under pathological conditions and stimuli, such as epilepsy, hypoxia, multiple sclerosis, bipolar, schizophrenia, H_2_O_2_, retinoids, and neuregulin-1^16,19–23^. Moreover, genetic studies implicate *Slc1a1*, a gene encoding EAAC1, in obsessive-compulsive disorder^24,25^.

The expression of EAAC1 varies during brain development^26^, and the transporter is expressed before both EAAT1 and EAAT2 expression in vitro^27,28^ and in vivo^29^. Early expression of EAAC1 suggests a role for EAAC1 in the neuroprotection of CNS cells during brain development.

Here, we show that EAAC1 is reduced or lost in NMS and EAAC1 (−/−) rats, respectively, in which nonselective attention-deficit and depressive behaviors were shown in adolescence. These results suggest that EAAC1 exerts a regulatory role in neuromodulation and that the reduction in EAAC1 expression contributes to the pathogenesis of depression.

## Results

### NMS rats exhibit impulsive behaviors in adolescence

**Fig. 1:**
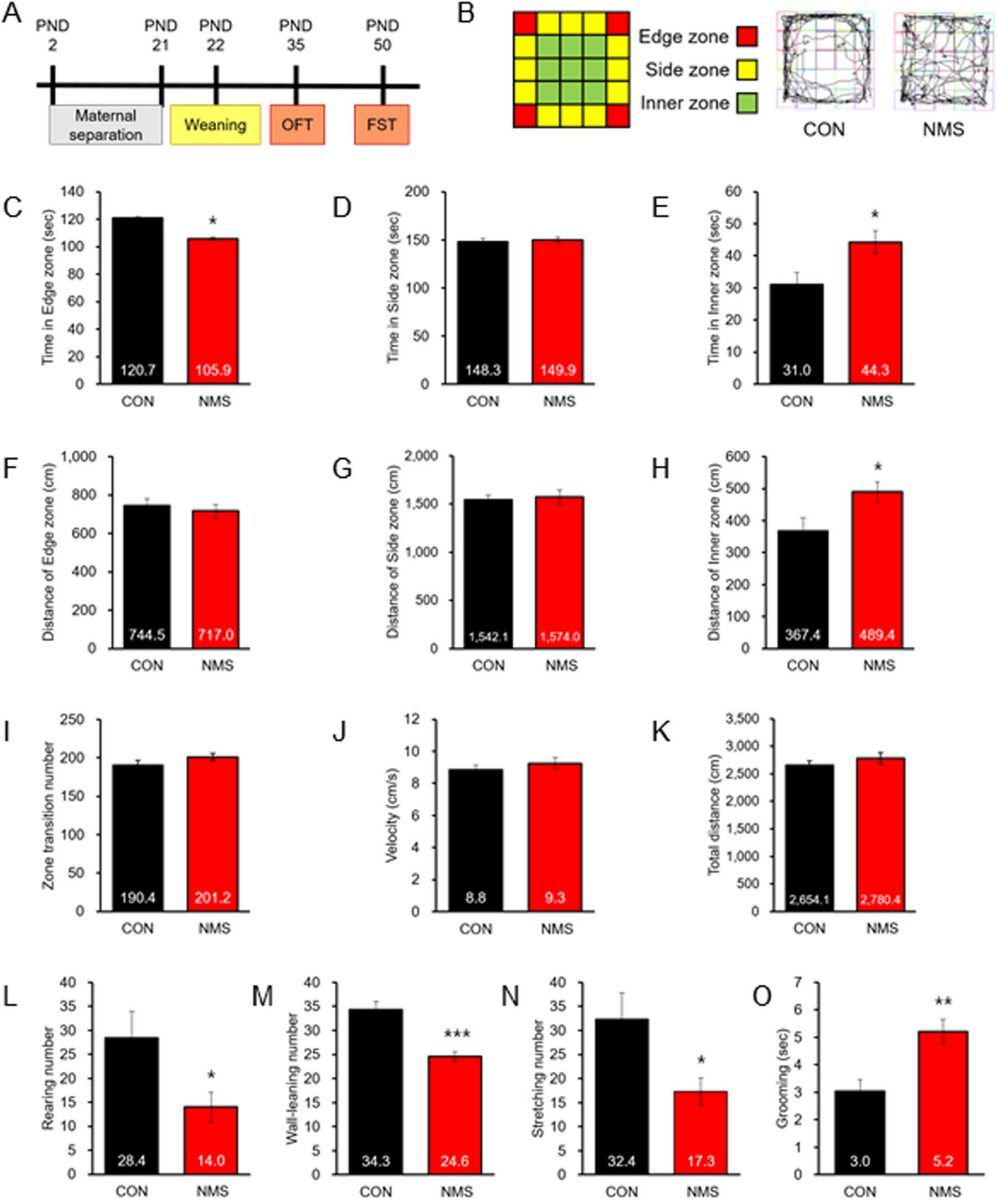
Schematic representation of the experimental schedule, exploratory, and ethological behavior. **a** Schematic representation of the experimental protocol. Maternal separation as a model of ELS was performed for 19 days (NMS21) from PND2 to 21. Pups were daily separated from the mother and siblings for 3 h/day and were placed individually in cages. Behavior tests were performed on PND 35 (OFT) and PND 50 (FST). **b** A square box on the left illustrates a schematic of the tested arena in an open field apparatus and shows areas that are designated as the edge, side, and inner zones. Representative track plots of a CON rat and NMS rat (on the left). Bar graph displaying the mean time spent in the edge zone (**c**), side zone (**d**), and inner zone (**e**). Bar graph displaying the distance of the edge zone (**f**), side zone (**g**), inner zone (**h**), and total traveled (**k**). Bar graph displaying the mean velocity (**j**) and zone transition number (**i**). **l** Number of rearings in OFT. **m** Number of wall-leanings in OFT. **n** Number of stretches in OFT. **o** Grooming time in OFT. NMS rats showed reduced rearing and wall-leaning within 5-min. The data on locomotion behavior quantification are expressed as the means ± S.E.M. The number of investigated rats is marked on each bar. **P < 0.01 and ***P < 0.001.

Several previous studies have shown that early-life stress alters emotional behavior in adults and adolescents. However, whether NMS leads to increased or decreased fearful/anxiety-like behaviors in adolescence remains unclear. In this study, we used an NMS model of daily separation from mother and siblings for 3 h/day individually (Fig. 1a).

In the first step, all the rats were investigated for their developmental milestones from PND1 to PND21 (additional file 2: Table S2a). When a certain developmental milestone (i.e., pinna detachment, incisor eruption, eye opening and surface right reflex) was measured, no significant difference was found between the NMS and CON groups (additional file 2: Table S2b, additional file 3: Fig. S1b). However, NMS rats showed a progressive decrease in body weight compared with CON rats (Additional file 3: Fig. S1a).

The open field apparatus comprises a square arena (5 × 5) divided into the edge zone, side zone, and inner zone on the captured image (Fig. 1b). When anxious, the natural tendency of rats is to prefer to stay closer to the wall or edge area. However, impulsive-related behavior is measured by the degree to which the rat prefers entering the inner zone of the open field. Comparison of the multiple parameters (such as the time and distance in the edge zone, side zone and inner zone) demonstrated that NMS rats exhibited increased impulsive-like behavior compared with CON rats (Fig. 1c–h). The NMS rats showed more exploration than CON in the inner zone (Fig. 1e, h). Furthermore, parameters such as the number of zone transitions, velocity of movement and total distance traveled were comparable between CON and NMS rats (Fig. 1i–k), revealing that NMS stress does not impair the general motor ability of adolescent rats.

### NMS rats exhibit decreased orienting behavior and increased grooming in adolescence

To verify these impulsive-like behaviors, we tested CON and NMS rats for orienting behavior in adolescence. As shown in Fig. 1l, m, NMS rats showed decreased rearing (CON, 28.4 ± 5.73, *n* = 21; NMS, 10.71 ± 2.00, *n* = 20; *t*(39) = 2.231, *P* < 0.05) and wall-leaning (CON, 34.33 ± 1.74, *n* = 21; NMS, 24.60 ± 0.96, *n* = 20; *t*(39) = 4.829, *P* < 0.001) in the open field. Moreover, compared with CON rats, NMS rats showed decreased stretching numbers (CON, 32.38 ± 5.42, *n* = 16; NMS, 17.30 ± 2.86, *n* = 14; *t*(28) = 2.362, *P* < 0.05; Fig. 1n). In contrast, NMS rats showed a significantly increased duration of self-grooming (CON, 3.04 ± 0.41, *n* = 15; NMS, 5.21 ± 0.45, *n* = 15; *t*(28) = 3.570, *P* < 0.01; Fig. 1o). These results indicate that NMS rats display deficits in nonselective attention behavior and self-grooming behavior.

### NMS rats provoke depressive behaviors in adolescence

**Fig. 2:**
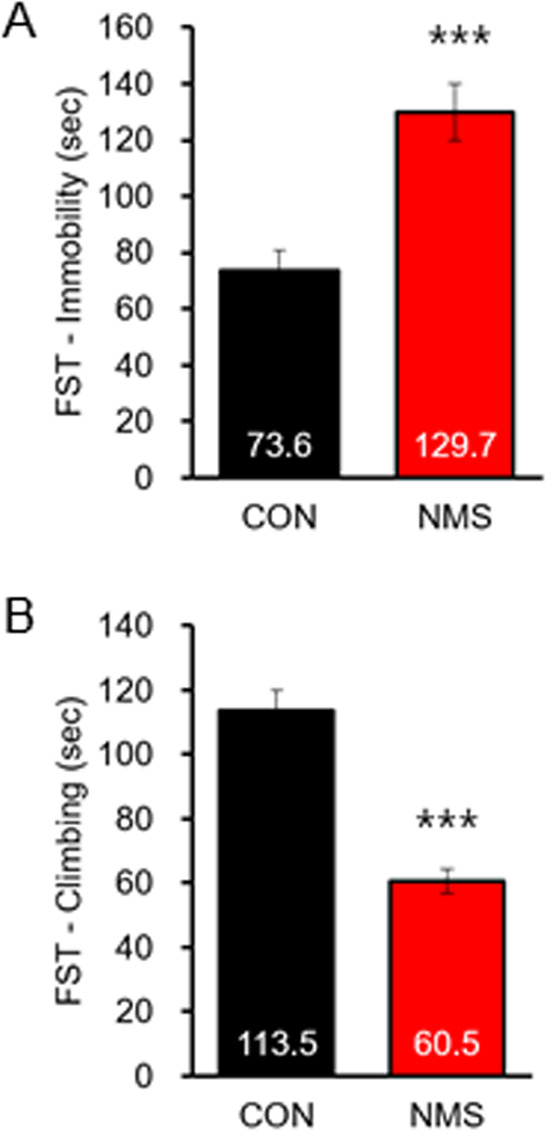
Effect of NMS adolescent rats on depressive-like behavior. NMS adolescent rats were submitted to the force swim test, and the immobility time (**a**) and climbing time (**b**) were measured. The data on depressive-like behavior quantification are expressed as the means ± S.E.M. The number of investigated rats is marked on each bar. ***P < 0.001.

We next analyzed the immobility and climbing time using the forced swimming test, which measures despair and depressive behaviors. We revealed that NMS induced depressive behaviors in adolescence. We found that NMS rats showed a significant increase in the duration of immobility (CON, 73.63 ± 7.16, *n* = 21; NMS, 129.74 ± 10.17, *n* = 24; *t*(43) = 4.391, *P* < 0.001; Fig. 2a). However, NMS rats showed significantly lower climbing than CON rats (CON, 113.47 ± 6.63, *n* = 21; NMS, 60.54 ± 3.83, *n* = 24; *t*(43) = 7.128, *P* < 0.001; Fig. 2b). These results indicate that NMS rats show depressive behaviors in adolescence.

### EAAC1 expression is altered in NMS rats of various lengths

**Fig. 3:**
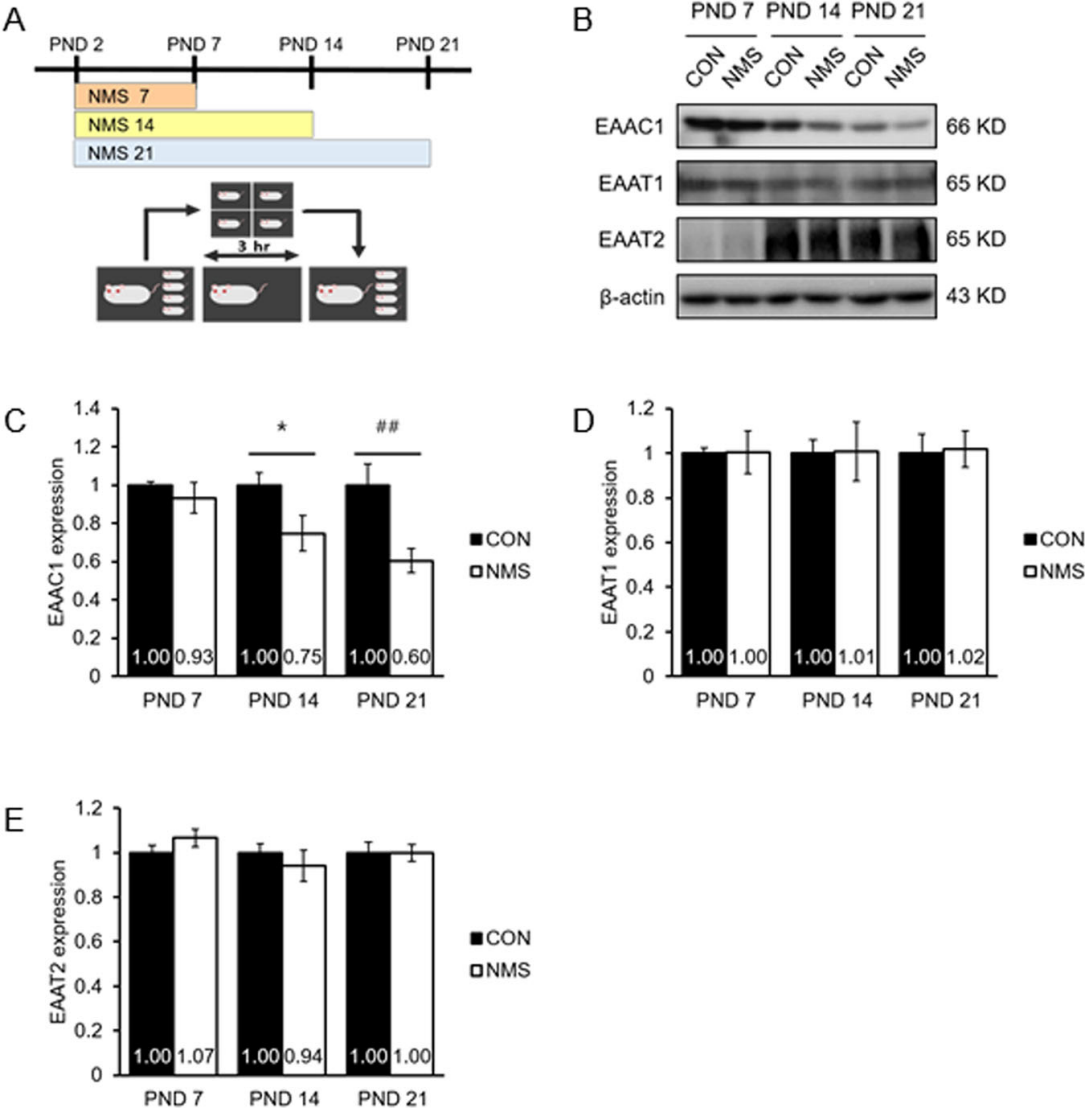
NMS downregulates EAAC1 expression in various duration NMS models. **a** Schematic representation of the experimental protocol. Maternal separation as a model of ELS was performed for 7 days (NMS7), 14 days (NMS14), and 21 days (NMS21). **b** Brain sampling was performed on each of the last days (7, 14, and 21 days). Immunoblot analysis of EAAC1, EAAT1, EAAT2, and β-actin expression in hippocampal lysates. **c** NMS attenuated the reduction in EAAC1 expression, as shown by the densitometric values, which are shown as ratios relative to the values of the CON group; CON *n* = 12, NMS *n* = 10 *P < 0.05, ^##^*P* < 0.01. **d** Quantification analysis of EAAT1 expression in (**b**). Ratios relative to the values of the CON group; CON *n* = 13, NMS *n* = 10. **e** Quantification analysis of EAAT2 expression in (**b**). Ratios relative to the values of the CON group; *n* = 10.

The glutamatergic circuit is involved in the pathogenesis of emotional behavior^30,31^. However, it remains unclear whether glutamate transporters are involved in impaired emotional behavior. Next, we performed NMS using three length models from PND 2 until PND 7, PND 14, or PND 21 (Fig. 3a). Some variability was revealed in the NMS lengths used. Western blotting was performed to examine the expression of EAAC1, EAAT1, and EAAT2 in the hippocampus (Fig. 3b). Interestingly, the level of the EAAC1 protein was significantly reduced in the length models of PND 14 and PND 21 (Fig. 3b, c). However, we observed no significant difference in the protein levels of EAAT1 and EAAT2 in all three length models (Fig. 3b–e). These results indicate that the expression of EAAC1 protein is easily affected by NMS stress from an early stage.

### EAAC1 expression is decreased in the ventral hippocampus of NMS rats in adolescence

**Fig. 4:**
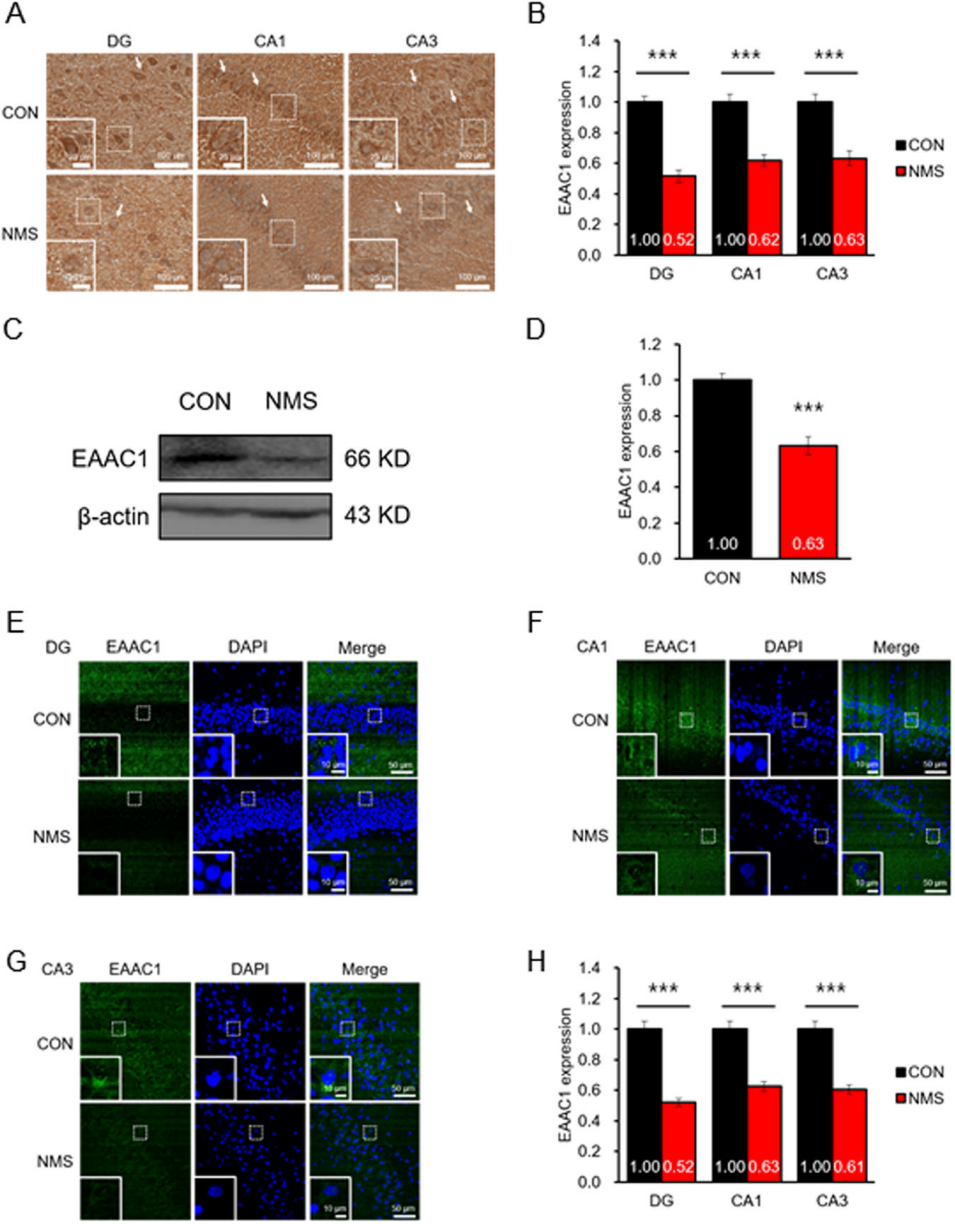
Expression of EAAC1 in the ventral hippocampus of NMS rats in adolescence. **a** Coronal sections of the ventral hippocampus of CON and NMS rats were stained with anti-EAAC1. Photomicrographs show the expression of EAAC1 in the DG, CA1, and CA3 regions of the ventral hippocampus in CON (top panels) and NMS (bottom panels) rats at PND50. Scale bar, 100 µm; inset, enlarged areas. Scale bar, 25 µm. **b** Quantification analysis of EAAC1 immunoreactivity in (**a**). Data on EAAC1 staining quantification are expressed as the means ± S.E.M.; *n* = 8. ***P < 0.001. **c** Immunoblot analysis of EAAC1 in the hippocampus of CON and NMS rats. **d** Quantification analysis of EAAC1 immunoreactivity in (**c**). The results are presented as the means ± S.E.M.; *n* = 12. ***P < 0.001. Representative image of EAAC1 expression in the DG (**e**), CA1 (**f**), and CA3 (**g**) regions of the ventral hippocampus. Scale bar, 50 µm; inset, enlarged areas. Scale bar, 10 µm. **h** Quantification analysis of EAAC1 immunoreactivity in (**e**–**g**). The data on EAAC1 staining quantification are expressed as the means ± S.E.M.; *n* = 11 (**e**), *n* = 12 (**f**), *n* = 12 (**g**). ***P < 0.001.

We next analyzed the expression of EAAC1 in the ventral hippocampus in adolescence. The ventral region is related to stress, emotion and affect^32^. The EAAC protein level was remarkably reduced in the ventral hippocampus of NMS rats (CON, 1.00 ± 0.04; NMS, 0.52 ± 0.04; *t*(20) = 8.933 in the DG, *P* < 0.001; CON, 1.00 ± 0.05; NMS, 0.62 ± 0.04; *t*(21) = 5.798 in the CA1; *P* < 0.001; CON, 1.00 ± 0.05; NMS, 0.63 ± 0.05; *t*(18) = 5.158 in the CA3; *P* < 0.001; Fig. 4a, b). Western blotting was performed to examine the expression of EAAC1 in the ventral hippocampus of NMS rats in adolescence. The EAAC1 protein level was significantly reduced in the ventral hippocampus of NMS rats in adolescence (Fig. 4c, d). Additionally, we performed immunofluorescence staining to visualize EAAC1 expression in the ventral hippocampus (CON, 1.00 ± 0.06; NMS, 0.52 ± 0.07; *t*(20) = 5.200 in the DG, *P* < 0.001; CON, 1.00 ± 0.03; NMS, 0.63 ± 0.08; *t*(22) = 4.374 in the CA1, *P* < 0.001; CON, 1.00 ± 0.04; NMS, 0.61 ± 0.08; *t*(22) = 4.486 in the CA3, *P* < 0.001; Fig. 4e–h). We confirmed that EAAC1 expression was decreased in the ventral hippocampus in NMS rats. Next, we checked the mRNA level of EAAC1 by performing mRNA microarray (additional file 4: Fig. S2a) and RT–PCR (additional file 4: Fig. S2b, c) in the ventral hippocampus of NMS rats in adolescence. We observed no difference in the mRNA level of EAAC1.

### EAAC1 expression is decreased in the cerebral cortex of NMS rats in adolescence

**Fig. 5:**
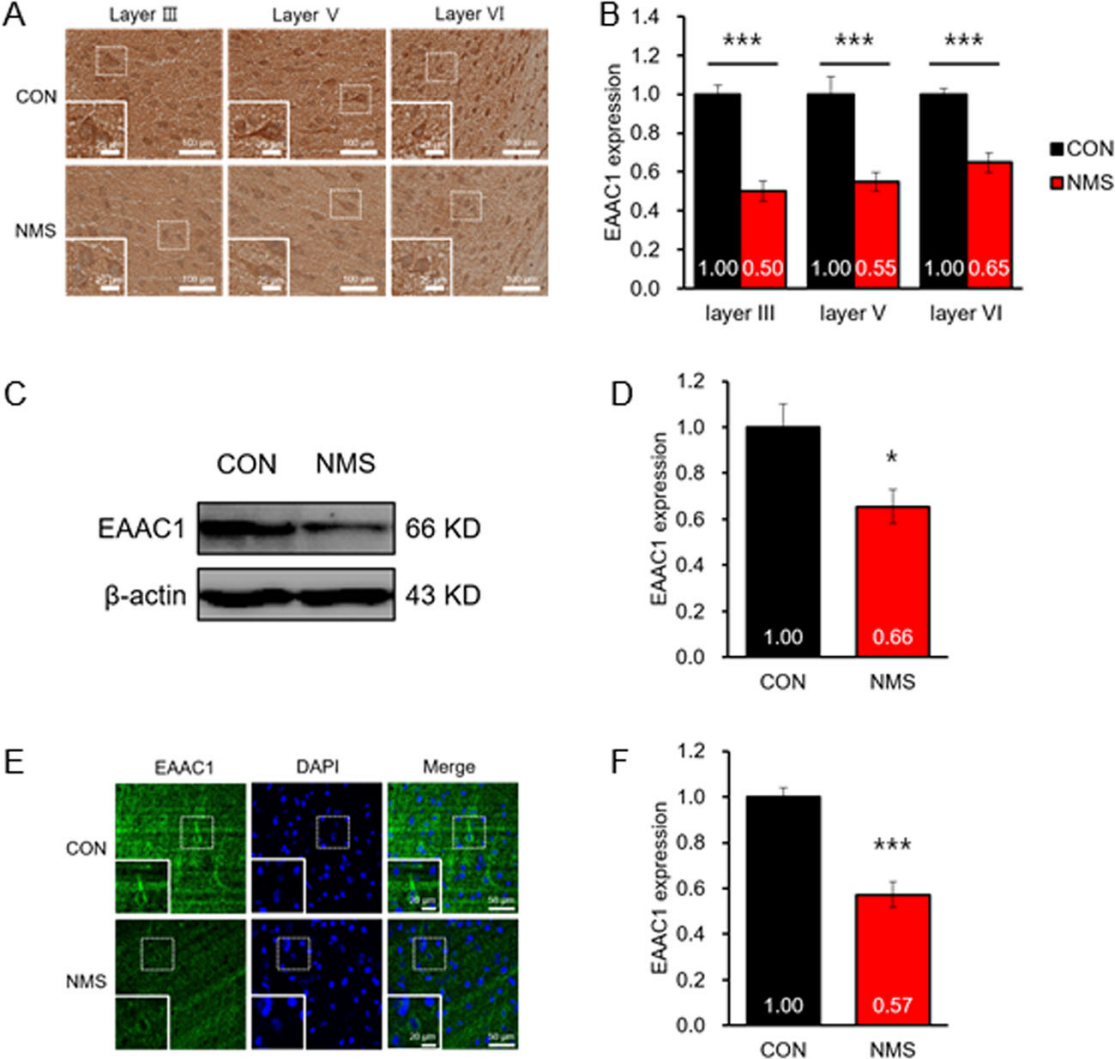
Expression of EAAC1 in the cortex of NMS rats. **a** Coronal sections of the cortex of CON and NMS rats were stained with anti-EAAC1. Photomicrographs show the expression of EAAC1 in layer III, layer V, and layer VI regions of the cortex in CON (top panels) and NMS (bottom panels) rats at PND50. Scale bar, 100 µm; inset, enlarged areas. Scale bar, 25 µm. **b** Quantification analysis of EAAC1 immunoreactivity in (**a**). The data on EAAC1 staining quantification are expressed as the means ± S.E.M.; CON *n* = 12, NMS *n* = 10 (layer III); CON *n* = 10, NMS *n* = 12 (layer V); CON *n* = 10, NMS *n* = 10 (layer VI). ***P < 0.001. **c** Immunoblot analysis of EAAC1 in the hippocampus of CON and NMS rats. **d** Quantification analysis of EAAC1 immunoreactivity in (**c**). The results are presented as the means ± S.E.M.; *n* = 15. *P < 0.05. **e** Representative image of EAAC1 expression in the cortex regions of the ventral hippocampus. Scale bar, 50 µm; inset, enlarged areas. Scale bar, 20 µm. **f** Quantification analysis of EAAC1 immunoreactivity in (**e**). The data on EAAC1 staining quantification are expressed as the means ± S.E.M.; CON *n* = 10, NMS *n* = 12. ***P < 0.001.

Similar to the ventral hippocampus, we stained the cerebral cortex sections in adolescence. We found that the EAAC1 protein level was remarkably reduced in the cerebral cortex of NMS rats (CON, 1.00 ± 0.05; NMS, 0.50 ± 0.05; *t*(18) = 7.222 in the layer III, *P* < 0.001; CON, 1.00 ± 0.09; NMS, 0.55 ± 0.05; *t*(20) = 4.641 in the layer V, *P* < 0.001; CON, 1.00 ± 0.03; NMS, 0.65 ± 0.05; *t*(20) = 6.256 in the layer VI, *P* < 0.001; Fig. 5a, b). Western blotting was performed to confirm the expression of EAAC1 in the cerebral cortex of NMS rats in adolescence. The EAAC1 protein level was significantly reduced in the cerebral cortex of NMS rats in adolescence (CON, 1.00 ± 0.10; NMS, 0.66 ± 0.08; *t*(28) = 2.745, *P* < 0.05; Fig. 5c, d). Furthermore, we performed immunofluorescence staining to visualize EAAC1 expression in the cerebral cortex (CON, 1.00 ± 0.04; NMS, 0.57 ± 0.06; *t*(20) = 5.962, *P* < 0.001; Fig. 5e, f).

### NMS induces a decrease in PV-positive GABAergic neurons and EAAC1 expression in the hippocampus of adolescence rats

**Fig. 6:**
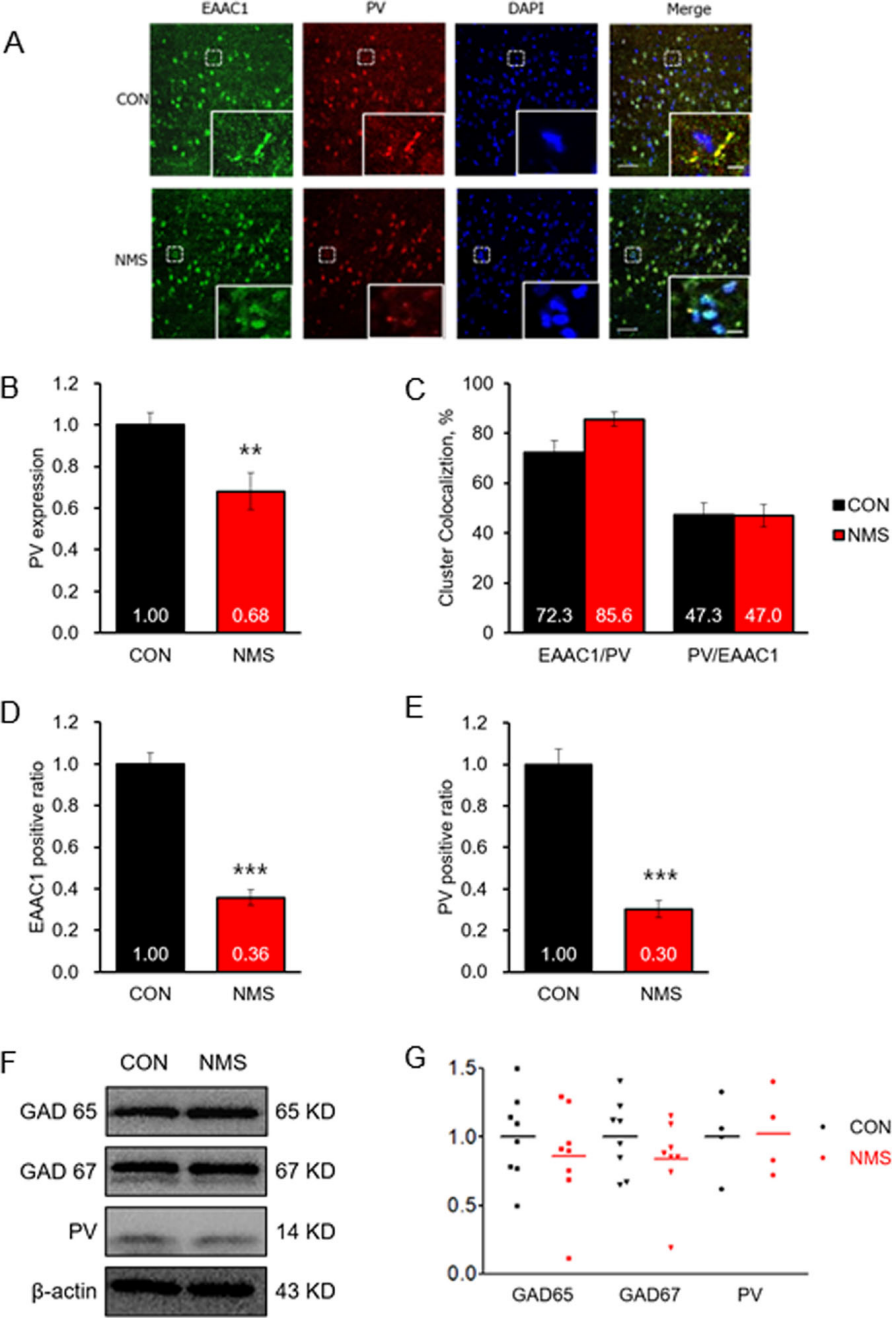
Decreased EAAC1 and PV-positive neurons in the hippocampus of NMS rats. **a** Coronal sections of the hippocampus were stained with anti-EAAC1 and anti-PV antibodies. Immunoreactivity was visualized using Alexa 488-conjugated secondary antibodies of anti-EAAC1 and Oyster 550 fluorescence-labeled primary antibodies of anti-PV. Scale bar, 50 µM; inset, enlarged areas. Scale bar, 5 µm. **b** Quantification analysis of PV immunoreactivity in (**a**). Fluorescence density of PV (red) and analysis by confocal microscopy. The data on PV staining quantification are expressed as the means ± S.E.M.; *n* = 10. **P < 0.01. **c** Quantification analysis of PV (red) clusters with EAAC1 (green) and EAAC1 clusters with PV in (**a**). The quantification data on EAAC1 and PV staining are expressed as the means ± S.E.M.; *n* = 16. **d** Quantification of the EAAC1 cluster number in (**a**). The results are presented as the means ± S.E.M.; *n* = 16. ***P < 0.001. **e** Quantification of the PV cluster number in (**a**). The results are presented as the means ± S.E.M.; *n* = 16. ***P < 0.001. **f** Immunoblot analysis of GAD65, GAD67, and PV in the hippocampus of CON and NMS rats. **g** Quantification analysis of GAD65, GAD67, and PV immunoreactivity in (**f**). The results are presented as the means ± S.E.M.; *n* = 8 (GAD 65, GAD67), *n* = 4 (PV).

We checked the immunoreactivity of EAAC1 and PV in NMS rats to investigate the involvement of the GABAergic pathway. Previously, we reported that EAAC1 is expressed in GABAergic neurons in the prefrontal cortex^16^. In further agreement, EAAC1 was found in PV-positive hippocampal interneurons of 3-week-old mice^15^. To determine the in vivo subcellular localization of EAAC1 in PV-positive neurons, we stained hippocampal sections of adolescent NMS rats. EAAC1 was detected in puncta-ring-like structures and neuropils, colocalizing with PV (Fig. 6a). Additionally, 72.34% of PV clusters were EAAC1-positive, suggesting EAAC1 localization at specific subsets of GABA terminals (Fig. 6c). However, 47.28% of EAAC1 clusters colocalized with PV (Fig. 6c), consistent with the notion that EAAC1 is also localized at non-GABAergic synapses^15^. Interestingly, NMS rats exhibit decreased expression of EAAC1 (CON, 1.00 ± 0.05; NMS, 0.36 ± 0.04; *t*(30) = 9.777, *P* < 0.001; Fig. 6d) and PV (CON, 1.00 ± 0.07; NMS, 0.30 ± 0.04; *t*(30) = 8.238, *P* < 0.001; Fig. 6e) at neuronal terminals. However, western blotting revealed that GAD65, GAD67, and PV did not significantly differ between the animal groups (Fig. 6f, g). Taken together, these results indicate that decreased EAAC1 expression in the PV-positive interneuron terminal may affect GABAergic dysfunction.

### EAAC1 (−/−) mice reduce orienting behavior in adolescence

**Fig. 7:**
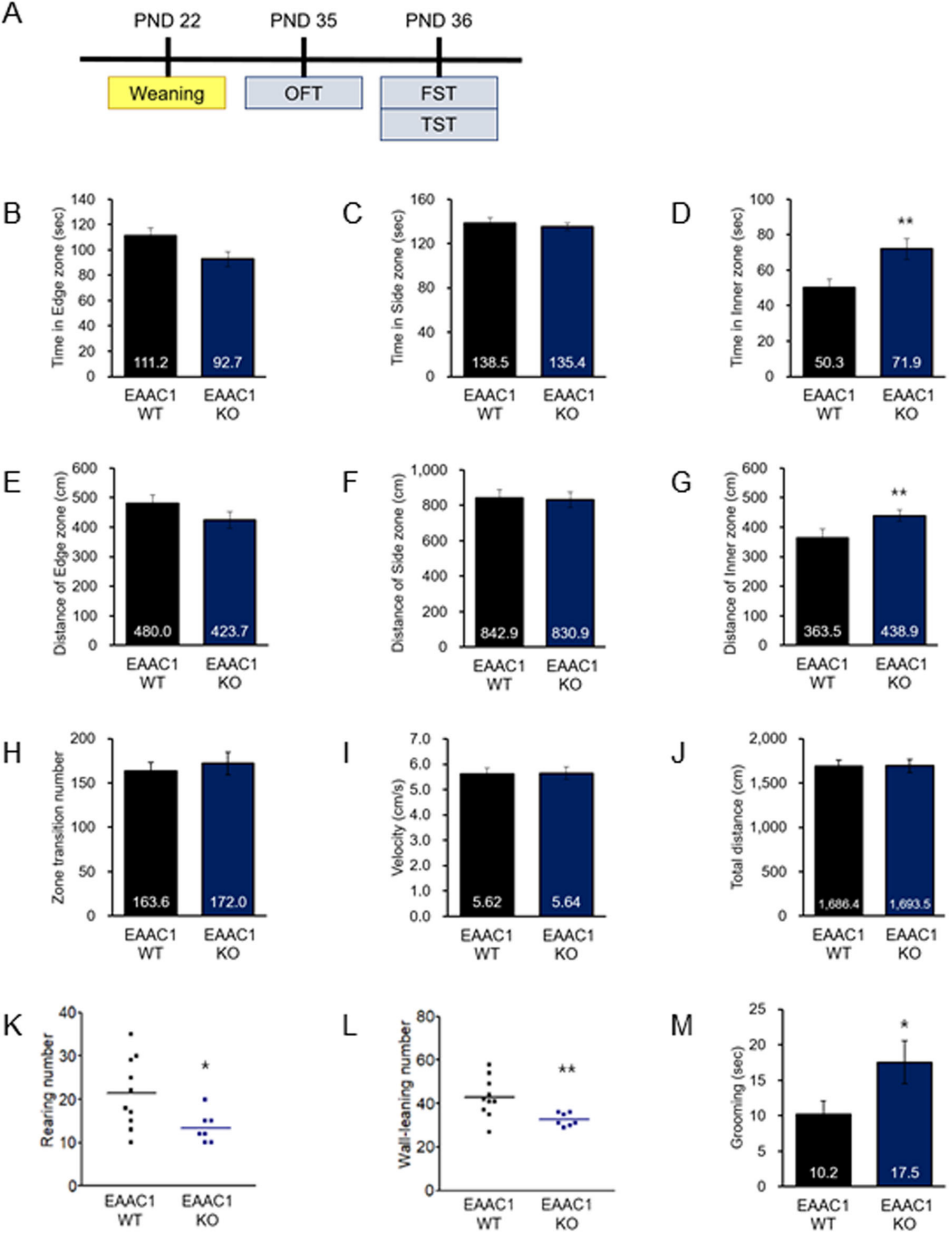
EAAC1 (−/−) mice exhibit impulsive-like behavior and reduce orienting behavior. **a** Behavior tests were performed on PND 35 (OFT), PND 36 (FST) and, PND 36 (TST). Bar graph displaying the mean time spent in the edge zone (**b**), side zone (**c**), and inner zone (**d**). Bar graph displaying the distance of the edge zone (**e**), side zone (**f**), and inner zone (**g**), as well as the total distance traveled (**h**). Bar graph displaying the mean velocity (**i**) and zone transition number (**j**). **k** Number of rearings in OFT. **l** Number of wall-leanings in OFT. **m** Grooming time in OFT. NMS rats showed reduced rearing and wall-leaning within 5 min. Data on locomotion behavior quantification are expressed as the means ± S.E.M. The number of investigated rats is marked on each bar. *P < 0.05, **P < 0.01.

We next assessed exploration in EAAC1 (−/−) mice because impulsive-like behavior was shown in NMS rats. Interestingly, EAAC1 (−/−) mice showed impulsive-like exploration, similar to NMS rats (EAAC1 WT, 50.33 ± 4.73, *n* = 20; EAAC1 (−/−), 71.92 ± 5.83, *n* = 15; *t*(33) = 2.94, *P* < 0.01; Fig. 7d) (EAAC1 WT, 363.53 ± 29.94, *n* = 20; EAAC1 (−/−), 483.886 ± 19.06; *t*(33) = 3.135, *n* = 15, *P* < 0.01; Fig. 7g). We then tested EAAC1 (−/−) mice for orienting behavior in adolescence. As shown in Fig. 7k, l, EAAC1 (−/−) mice showed decreased rearing (EAAC1 WT, 21.40 ± 2.45; EAAC1 (−/−), 13.43 ± 1.24; *t*(15) = 2.543, *P* < 0.05) and wall-leaning (EAAC1 WT, 42.80 ± 2.73; EAAC1 (−/−), 32.57 ± 1.05; *t*(15) = 2.998, *P* < 0.01) in the open field. EAAC1 (−/−) mice showed an increased duration of self-grooming (EAAC1 WT, 10.16 ± 1.92; EAAC1 (−/−), 17.53 ± 3.00; *t*(33) = 2.163, *P* < 0.05) (Fig. 7m), corroborating the results of a previous report^33^. These results indicate that EAAC1 (−/−) mice display deficits in orienting and nonselective attention behavior.

### EAAC1 (−/−) mice exhibit depressive behaviors in adolescence

**Fig. 8:**
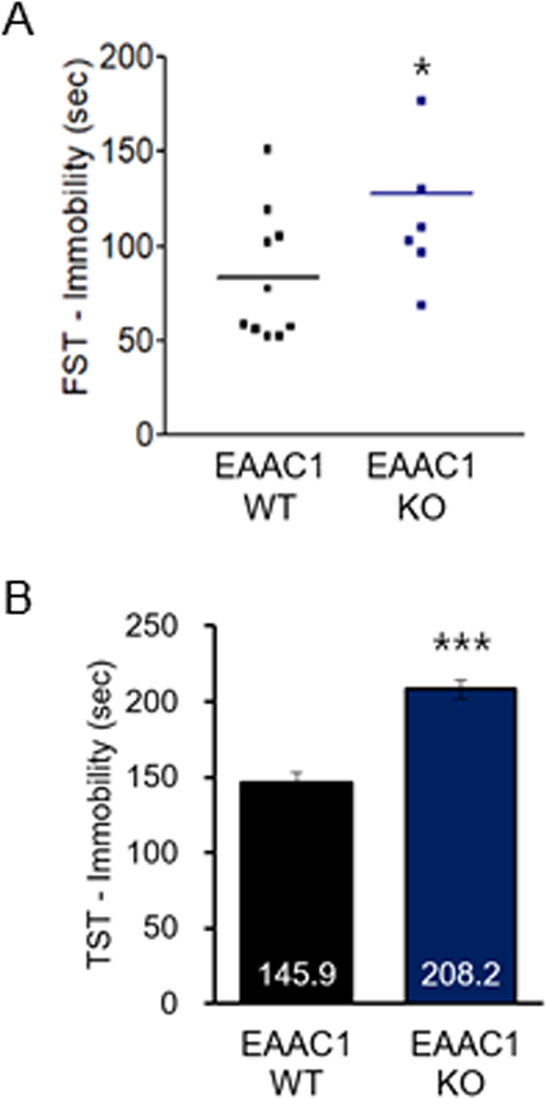
EAAC1 (−/−) mice show depressive-like behavior in adolescence. **a** EAAC1 (−/−) mice were submitted to the force swim test, and the immobility time was measured. **b** EAAC1 (−/−) mice were subjected to the tail suspension test, and the immobility time was measured. The data on depressive-like behavior quantification are expressed as the means ± S.E.M.; *n* = 10. *P < 0.05, ***P < 0.001.

EAAC1 (−/−) mice showed an increased time of immobility using the forced swimming test (EAAC1 WT, 83.13 ± 10.34 *n* = 10; EAAC1 (−/−), 127.40 ± 18.34, *n* = 7; *t*(15) = 2.208, *P* < 0.05; Fig. 8a). Therefore, we monitored the immobility time using the tail-suspension test, which also measures despair and depressive behaviors. We found that EAAC1 (−/−) mice showed a significant increase in the duration of immobility (EAAC1 WT, 145.91 ± 7.36, *n* = 14; EAAC1 (−/−), 208.15 ± 16.09, *n* = 11; *t*(23) = 6.275, *P* < 0.001; Fig. 8b, additional file 5: Video S1). We revealed for the first time that EAAC1 (−/−) mice show depressive-like behaviors in adolescence.

## Discussion

Early life is a sensitive period of brain development where experiencing any insult (such as stressful events) can cause far-reaching consequences. ELS has been established as a major risk factor for major depression and suicidal behavior along with other psychiatric illnesses in adolescence and adulthood^1,34–36^. The NMS model is a well-established animal model of early-life stress. However, different behavioral phenotypes have been reported depending on various NMS protocols, such as a single or sibling’s separation, the duration of separation, and the number of separations. In adolescence, increased and decreased fearful/anxiety-related behaviors, increased and no effect on depressive-like behaviors were all reported^37,38^. We used transient NMS during the first three postnatal weeks as well as characterized the model of early-life stress in adolescence.

In the present study, we found that EAAC1 protein expression was significantly decreased in the hippocampus and cerebral cortex of adolescent NMS rats. However, NMS did not affect the mRNA level of EAAC1 in the hippocampus in adolescence. These results may indicate regulation of the EAAC1 protein degradation pathway. Additionally, we did not observe significantly reduced EAAT1 and EAAT2 expression in the hippocampus of adolescent NMS rats. Previous human postmortem studies reported reduced expression of EAAC1 (EAAT3) and EAAT4 mRNA in the striatum in bipolar disorder^39^. Prenatal restraint stress offspring rats showed significantly induced depressive-like behavior and decreased EAAT2 and EAAT3 mRNA expression in the hippocampus^40^. In contrast, the transcript levels of EAAC1 were not significantly altered in learned helpless rats, an animal model of depression^41^.

Our data revealed that male NMS rats showed increased impulsive behavior in adolescence. During the open field task, male NMS rats spent a significantly longer time exploring a longer distance in the inner zone (Fig. 1). These findings are in agreement with the previously reported decreased anxiety-like behavior after experiencing maternal separation during the first two postnatal weeks^42^. Male NMS rats spent less time in the periphery (edge and side zone), suggesting that they were more impulsive than CON rats^43^. Moreover, we found for the first time that NMS rats showed hyperactivity only in the inner zone. Rats normally prefer to stay beside the wall where no predators may attack from the back. Furthermore, we found that EAAC1 (−/−) mice exhibited impulsive-like behavior as NMS rats. The initially reported *Slclal*/EAAT3-null mice showed decreased activity in the open field^44^, whereas other investigators have reported no significant changes in EAAC1 (−/−) mice^45,46^.

Rearing consists of stopping ambulation and standing on the hindlimbs and is used as a measure of orienting behavior because orienting or nonselective attention is associated with the duration of rearing episodes, where longer rearing indicates more orienting behavior^38^. Both rearing and leaning (against the walls) to novelty have been associated with the distribution of hippocampal mossy fibers in rats^47,48^. Additionally, rearing activity was differentially affected by hippocampal cholinergic and dynorphinergic systems, inhibition of nitric oxide synthesis, and blockade of NMDA glutamate receptors^49–51^. The NMS rats showed significantly less rearing and wall-leaning episodes than CON rats, indicating that they may have a deficit in nonselective attention and orienting. Here, we report that EAAC1 (−/−) mice also showed decreased rearing and wall-leaning activity.

Moreover, stretch attend postures occur a risk-assessment behavior indicating that the rodent is hesitant to move from its present location to a new position^52,53^; thus, a high frequency of these postures indicates a higher level of anxiety. We observed that NMS rats showed decreased stretching numbers in adolescence. These findings correlate with an increase in spending time in the inner zone; NMS rats showed a decrease in spending time in the edge zone.

Acute stressors, including exposure to a novel environment, can potently modulate self-grooming, often increasing the frequency or total duration of episodes^54–56^. Our data revealed increased self-grooming behavior in NMS rats and EAAC1 (−/−) mice in adolescence. Interestingly, several independent genetic studies consistently implicate the Slc1al gene, which encodes EAAC1, a candidate gene for obsessive-compulsive disorder in both males and females^57–59^. The sequential patterned strokes performed during grooming are reminiscent of the excessive and repetitive hand-washing behaviors of patients with OCD^60^. Regarding the different grooming behavior phenotypes, *Slc1al*/EAAC1-null mice demonstrated no significant changes in 8- to 16-wk-old mice^45^, and others have reported increased grooming activity in EAAC1 knock-out mice (P14-P35)^33^. Additionally, other reports have published evidence for a role of EAAC1 in schizophrenia. Increased *Slc1al*/EAAC1 transcripts and proteins were reported in schizophrenic subjects^21^. Interestingly, we previously found that NRG1/ErbB4 (susceptibility genes of schizophrenia) signaling is critical for EAAC1 expression and function^16^.

Our data showed that NMS rats provoke depressive-like behavior in adolescence. Notably, EAAC1 (−/−) mice also showed depressive-like behavior in adolescence. Adolescence correlates with developmental changes in the brain circuits in the limbic areas, such as the hippocampus^61^. Some investigations have demonstrated that applying pharmacological treatments during adolescence has beneficial effects on the mitigation of depressive-like behaviors induced by early-life stress^62^. However, no previous studies have investigated EAAC1 in early-life stress circuits or concerning depressive-like behaviors. Here, we reported that EAAC1 (−/−) mice significantly show depressive-like behavior in adolescence. Moreover, we found that EAAC1 expression was reduced in PV-positive hippocampal GABAergic neurons in the NMS group. Depression and stress decrease both glutamate and GABA neurotransmitter circuits in the limbic and cortical regions^63,64^. The loss of EAAC1 expression may cause an abnormality in glutamate conversion to GABA in presynaptic GABAergic terminals. Further research is needed to clarify the underlying mechanism of EAAC1 in these phenotypes. EAAC1 can transport not only extracellular glutamate but also cysteine into the neurons.

Our data clarified that early-life stress, such as NMS, can provoke impulsive-like behavior, orienting and nonselective attention deficit, abnormal grooming behavior, and depressive-like behaviors in adolescence. Furthermore, we identified a novel function of EAAC1: EAAC1 (−/−) mice show similar abnormal behaviors to NMS in adolescence, which may be associated with decreased EAAC1 expression. These results suggest that early-life stress can cause changes in EAAC1 expression, which may participate in the pathogenesis of nonselective attention deficit and depression in adolescence. These results may provide new insights into EAAC1 signaling in the pathophysiology of depression and shed light on the development strategies of new antidepressants.

## Materials and methods

### Reagents and antibodies

Antibodies were supplied by Santa Cruz Biotechnology Inc. (Santa Cruz, CA, USA) (EAAT1, sc-15316; EAAT3, sc-25658; GAD67, sc-58531; mouse IgG, sc-2025; rabbit IgG, sc-66931; β-actin, sc-47778; HRP-conjugated anti-rabbit IgG, sc-2004; HRP-conjugated anti-mouse IgG, sc-2005 and HRP-conjugated anti-goat IgG, sc-2020), Cell Signaling Technology (CST, MA, USA) (EAAT2, #3838), Abcam (Cambridge, UK) (parvalbumin (PV), ab11427), Synaptic System (Gottingen, Germany) (PV, 195011C3), Sigma (St. Louis, MO, USA) (GAD65, G1166), and Millipore Corporation (Chemicon, MA, USA) (EAAT3(EAAC1), MAB1587).

### Animals and maternal separation procedure

Experiments with animals were performed following the institutional and Eulji University guidelines. Pregnant Sprague-Dawley (SD) rats were obtained at 15–17 days of gestation from a laboratory animal supplier (Samtako bio Korea) and were housed individually in cages under standard laboratory conditions with a 12-h light/12-h dark cycle. On postnatal day (PND) 1, the litters were culled to 6–10 pups at a constant sex ratio of 1:1 to avoid sex-based maternal behavioral biases^65,42^. During separation sessions, the pups were removed from the mother for periods of 3 h per day. From PND1, the pups were weighed daily, and their overall health was assessed (i.e., milk visible in pup’s stomach and pups age-appropriately active). Daily, each pup was assessed for the achievement of species-specific developmental milestones as described in Additional file 2: Table S2^66^. EAAC1 (−/−) mice were also housed in a regulated environment. EAAC1 (−/−) mice were descendants of the strain established by Peghinni et al.^44^, in which exon 1 was disrupted by a neomycin resistance cassette. EAAC1 (−/−) mice were outbred to wild-type (WT) CD1 mice for more than 10 generations prior to these studies. A WT colony was maintained using the WT offspring from the latter outcrosses. We used only males for this experiment.

### Statistical analysis

The data are presented as the means ± SEM of three or more independent experiments. Student’s paired *t*-test was used for comparisons of the means between two groups of cells in a single experiment. *P* < 0.05 was considered statistically significant.

### Supplemental materials and methods

The Supplemental Materials and Methods are provided in additional file 1: Table S1.

## Supplementary information

Additional files figure legends

Additional file 1 : Table S1

Additional file 2. Table S2

Additional file 3 : Figure S1

Additional file 4 : Figure S2

Additional file 5 : Video 1
