# Correction: Early-life stress induces EAAC1 expression reduction and attention-deficit and depressive behaviors in adolescent rats

**DOI:** 10.1038/s41420-020-00353-4

**Published:** 2020-11-10

**Authors:** Han-Byeol Kim, Ji-Young Yoo, Seung-Yeon Yoo, Sang Won Suh, Seoul Lee, Ji Hye Park, Jun-Ho Lee, Tai-Kyoung Baik, Hye-Sun Kim, Ran-Sook Woo

**Affiliations:** 1 Department of Anatomy and Neuroscience, College of Medicine, Eulji University, Daejeon, 34824 Republic of Korea; 2 Department of Physiology, College of Medicine, Hallym University, Chuncheon, 24252 Republic of Korea; 3 Department of Pharmacology and Brain Research Institute, College of Medicine, Wonkwang University, Jeonbuk, 54538 Republic of Korea; 4 Department of Emergency Medical Technology, Daejeon University, Daejeon, 34520 Republic of Korea; 5 Department of Pharmacology, College of Medicine, Seoul National University, Seoul, 110-799 Korea; 6 Seoul National University College of Medicine, Bundang Hospital, Sungnam, 13620 Republic of Korea

**Keywords:** Depression, Molecular neuroscience

Correction to: *Cell Death Discovery*

10.1038/s41420-020-00308-9

published online 08 August 2020

The original version of this article contained an error in the name of one of the co-authors (Sang Won Suh). This has been corrected in the PDF and HTML versions.

