# Supplementary material for: Early-life stress induces EAAC1 expression reduction and attention-deficit and depressive behaviors in adolescent rats
Source: Cell Death Discov. 2020 Aug 8;6:73. doi: 10.1038/s41420-020-00308-9 (PMC7415155; doi:10.1038/s41420-020-00308-9)
Supplement: Supplementary file 1 — Additional files figure legends [file 41420_2020_308_MOESM1_ESM.docx]

**Additional files**

**Additional file 1: Table S1.** Supplemental materials and methods.

**Additional file 2: Table S2.** Assessment of developmental milestones in pups

**Additional file 3: Fig. S1.** Average body weights and surface right reflex

Daily performance of the body weight (**a**) and surface right reflex (**b**) of CON and NMS pups. The results are presented as the means ± S.E.M.; PND 21: CON, 61.33±2.00, n=17; NMS, 55.00±0.47, n=17. **P*<0.05.

**Additional file 4: Fig. S2.** Expression levels of EAAC1 mRNA

**a**, RNA microarray was observed in NMS rats, n=3. **b**, RT–PCR was performed to examine EAAC1 expression in NMS rats. **c**, Quantification analysis of data in **b**, n=6.

**Additional file 5: Video S1.** EAAC1 (-/-) mice show depressive-like behavior in TST
