# Supplementary material for: Early-life stress induces EAAC1 expression reduction and attention-deficit and depressive behaviors in adolescent rats
Source: Cell Death Discov. 2020 Aug 8;6:73. doi: 10.1038/s41420-020-00308-9 (PMC7415155; doi:10.1038/s41420-020-00308-9)
Supplement: Supplementary file 2 — Additional file 1 : Table S1 [file 41420_2020_308_MOESM2_ESM.docx]

**Additional file 1. Table S1**

**Supplemental Materials and Methods**

***Behavioral analysis***

The behavior of NMS and CON rats was investigated in parallel. To minimize the influence of circadian rhythms, all behavioral tests were conducted during the same phase of the circadian cycle (i.e., light phase). One investigator performed all behavioral testing to prevent interobserver variability due to different handling of pups. The exploratory and depressive-like behaviors of CON and NMS rats were tested in adolescence (P36-50).

***Exploratory behavior in the open field***

The open field test (OFT) was performed as previously reported ^35, 36^. The open field testing arena (rat, 60 cm × 60 cm × 30 cm; mouse, 30 cm × 30 cm × 25 cm) was divided into 25 squares using a computer tracking system (SMART® version 3.0.05; Panlab Harvard Apparatus, Barcelona, Spain). The nine squares in the central area were defined as the inner zone, the four squares at each corner were defined as the edge zone, and the remaining squares in the periphery were defined as the side zone. Adolescent rats (PND36-PND50) and EAACl (-/-) (PND35-PND36) mice were allowed to freely explore the testing arena for 5 min. The running distance by the rats in the edge, side, and inner zones, as well as the spending time, zone transition number, and velocity, were quantified. Additionally, the occurrence and duration of wall-rearing, stretching, and grooming were analyzed.

***Forced swimming test (FST)***

The FST was conducted as described previously ^37^. After the OPT, the same animals were subjected to the FST on PND 50. Each rat was allowed to swim in an acrylic cylinder (20-cm diameter; 45-cm height) filled with water to a 30-cm depth (23-25°C). On PND 50, the rats were individually placed into the cylinder containing water for 15 min as pre-swim trials, and then they were carefully dried and returned to in their house cage. On the following day, the rats were subjected to the FST for 5 min (test trial), and the immobility and climbing times were recorded in seconds. Between every subject, the water was removed and refilled again to avoid any smell trail. Each mouse was allowed to swim in an acrylic cylinder (10-cm diameter; 45-cm height) filled with water to a 15-cm depth.

***Tail suspension test (TST)***

The test was performed as previously described ^38^. The mice were individually suspended by the tail; to prohibit climbing, climbstoppers were placed around their tails before applying adhesive tape. The duration of immobility was measured for 6 min and defined as the time when the mice were completely motionless and hung passively.

***RNA microarray analysis and RT–PCR***

Total RNA was isolated from cells using TRIzol (Invitrogen). RNA quality was assessed using an Agilent 2100 Bioanalyzer and RNA 6000 Nano Chip system (Agilent Technologies, Amstelveen, The Netherlands), and RNA quantification was performed using an ND-2000 Spectrophotometer (Thermo Inc., DE, USA). For the control (CON) and test RNAs, library construction was performed using a QuantSeq 3’mRNA-Seq Library Prep Kit (Lexogen, Inc., Austria) according to the manufacturer’s instructions. Briefly, each 500 ng total RNA was prepared, an oligo-dT primer containing an Illumina-compatible sequence at its 5’end was hybridized to the total RNA, and then reverse transcription was performed. After degradation of the RNA template, second-strand synthesis was initiated by a random primer containing an Illumina-compatible linker sequence at its 5’end. The double-stranded library was purified by using magnetic beads to remove all reaction components. The library was amplified to add the complete adapter sequences required for cluster generation. The completed library was purified from the PCR components. High-throughput sequencing was performed as single-end 75-bp sequences using NextSeq 500 (Illumina Inc., USA). cDNA synthesis was performed using an iScript™ cDNA Synthesis Kit (Bio-Rad Laboratories, Inc., USA; cat. no: 170-8891). The following primers were used for PCR: slc1a1 (F): 5’-CT TCT GTC CTC ATC CTG TAA AT-3’, (R): 5’-TA AGA TGA CTT AAG GGC ACT AC-3’ and GAPDH (F): 5’-TGT GAG GGA GAT GCT CAG TG-3’ (R): 5’-GTG GAC CTC ATG GCC TAC AT-3’. All the primers were designed using primer 3 programs according to the known or predicted rat sequences reported in GenBank. PCR was performed using Maxime PCR PreMix (i-Taq) (iNtRON Biotechnology, Inc., Korea; cat. no: 25026). For all of the probands, each cycle consisted of a denaturation step at 95 ºC for 10 sec, followed by separate annealing (20 sec) and extension (30 sec) steps at a temperature that was characteristic for each proband.

***Data acquisition and analysis****.*

QuantSeq 3’mRNA-Seq reads were aligned using Bowtie2 ^39^. Bowtie2 indices were either generated from the genome assembly sequence or representative transcript sequences to align to the genome and transcriptome. The alignment file was used to assemble transcripts, estimate their abundances and detect the differential expression of genes. Differentially expressed genes were determined based on counts from unique and multiple alignments using coverage in Bedtools ^40^. The RC (read count) data were processed based on the quantile normalization method using EdgeR within R (R Development Core Team, 2016) and Bioconductor ^41^. Gene classification was based on searches performed by DAVID (http://david.abcc.ncifcrf.gov/) and Medline databases (<http://www.ncbi.nlm.nih.gov/>).

***Western blot analysis***

Western blotting was carried out as previously described ^42^. Briefly, tissues were resolved using a modified homogenizing buffer (50 mM Tris-HCl [pH 7.4], 150 mM NaCl, 1% NP-40, 0.25% sodium-deoxycholate, 1 mM PMSF, 1 mM EDTA, and 1 μg/ml each of aprotinin, leupeptin, and pepstatin protease inhibitors). The samples were then resolved using SDS-PAGE and were transferred to nitrocellulose membranes, followed by blocking with TBS containing 5% fat-free milk and 0.05% Tween 20 for 1 hr. Thereafter, the membranes were incubated overnight at 4°C with primary antibodies and were developed using horseradish peroxidase-conjugated secondary antibodies and an enhanced chemiluminescence system (Amersham Pharmacia).

***Immunofluorescence analysis***

Immunofluorescence of rat brain sections was performed as previously described ^17^. Briefly, cryosections (15 µm) were fixed with 4% paraformaldehyde in PBS for 20 min. After fixation, the sections were permeabilized for 10 min with 0.5% Triton X in PBS. The sections were incubated overnight at 4°C in PBS containing mouse anti-EAAC1 (1:100) and anti-PV (1:200), followed by incubation with Alexa Fluor^®^488 goat anti-mouse IgG (Jackson ImmunoResearch Laboratories, Inc., 1:200) in buffer for 2 hr at room temperature. The nuclei were counterstained with DAPI (10 μM in PBS) for 30 min. The stained cells were mounted in Vectorshield (Vector Laboratories) and were observed under an LSM 5 LIVE confocal microscope (Carl Zeiss AG, Oberkochen, Germany).

***Immunohistochemistry analysis***

To retrieve antigenicity, dewaxed sections were boiled within 0.1 mol/L of citrate-buffered saline (pH 6.0) for 10 min. After cooling for 30 min, the sections were rinsed in PBS. After fixation, the endogenous peroxidase was quenched by 1% hydrogen peroxide in 10% methanol for 30 min. After two washes in PBS-T (0.2% Triton X-100 in 0.1 mol/L PBS, pH 7.6) for 5 min each, the sections were blocked for 1 hr in blocking solution (5% host serum + 1% BSA in PBS-T) and incubated in primary antibody (anti-EAAC1, 1:200) at 4°C overnight. After washing with PBS-T, the sections were incubated with a biotinylated secondary antibody for 1 hr at RT. After PBS rinsing and avidin-biotin-peroxidase complex (Vectastain Elite ABC kit) treatment for 1 hr at RT, the sections were developed for 5 min in a 0.05% DAB solution. The stained cells were mounted in Vectorshield (Vector Laboratories) and were observed under an LSM 5 LIVE confocal microscope (Carl Zeiss AG, Oberkochen, Germany).
