# Supplementary material for: Early-life stress induces EAAC1 expression reduction and attention-deficit and depressive behaviors in adolescent rats
Source: Cell Death Discov. 2020 Aug 8;6:73. doi: 10.1038/s41420-020-00308-9 (PMC7415155; doi:10.1038/s41420-020-00308-9)
Supplement: Supplementary file 3 — Additional file 2. Table S2 [file 41420_2020_308_MOESM3_ESM.docx]

| **Developmental assessment** | **Criteria** | **PND** |
| --- | --- | --- |
| Somatic development | | |
| Body weight |  | PND 1 - 21 |
| Pinna detachment | Both pinnas detached completely | PND 1 - 3 |
| Incisor eruption | All four incisors erupted | PND 7 - 12 |
| Eye opening | Both eyes open completely | PND 12 - 15 |
| Behavioral development | | |
| Surface right reflex | Time to right with all four paws on surface | PND 3 - 9 |

**Additional file 2. Table S2**

A

B

| **Assessment** | | **CON (%)** | **NMS(%)** | **X²** | **p** |
| --- | --- | --- | --- | --- | --- |
| Pinna | PND1 | 23.53 | 5.88 | 0.26 | 0.16 |
|  | PND2 | 82.35 | 76.47 | 0.03 | 0.68 |
|  | PND3 | 100.00 | 100.00 | 0.00 | n.s |
| Incisor | PND7 | 0.00 | 0.00 | 0.00 | n.s |
|  | PND8 | 8.33 | 25.00 | 0.17 | 0.29 |
|  | PND9 | 41.67 | 66.67 | 0.38 | 0.24 |
|  | PND10 | 75.00 | 66.67 | 0.04 | 0.67 |
|  | PND11 | 83.33 | 91.67 | 0.04 | 0.56 |
|  | PND12 | 100.00 | 100.00 | 0.00 | n.s |
| eye | PND12 | 8.33 | 0.00 | 0.04 | 0.33 |
|  | PND13 | 33.33 | 8.33 | 0.37 | 0.14 |
|  | PND14 | 66.67 | 66.67 | 0.00 | 1.00 |
|  | PND15 | 100.00 | 100.00 | 0.00 | n.s |

Table S2.
