# Supplementary figures and images for: Early-life stress induces EAAC1 expression reduction and attention-deficit and depressive behaviors in adolescent rats
Source: Cell Death Discov. 2020 Aug 8;6:73. doi: 10.1038/s41420-020-00308-9 (PMC7415155; doi:10.1038/s41420-020-00308-9)

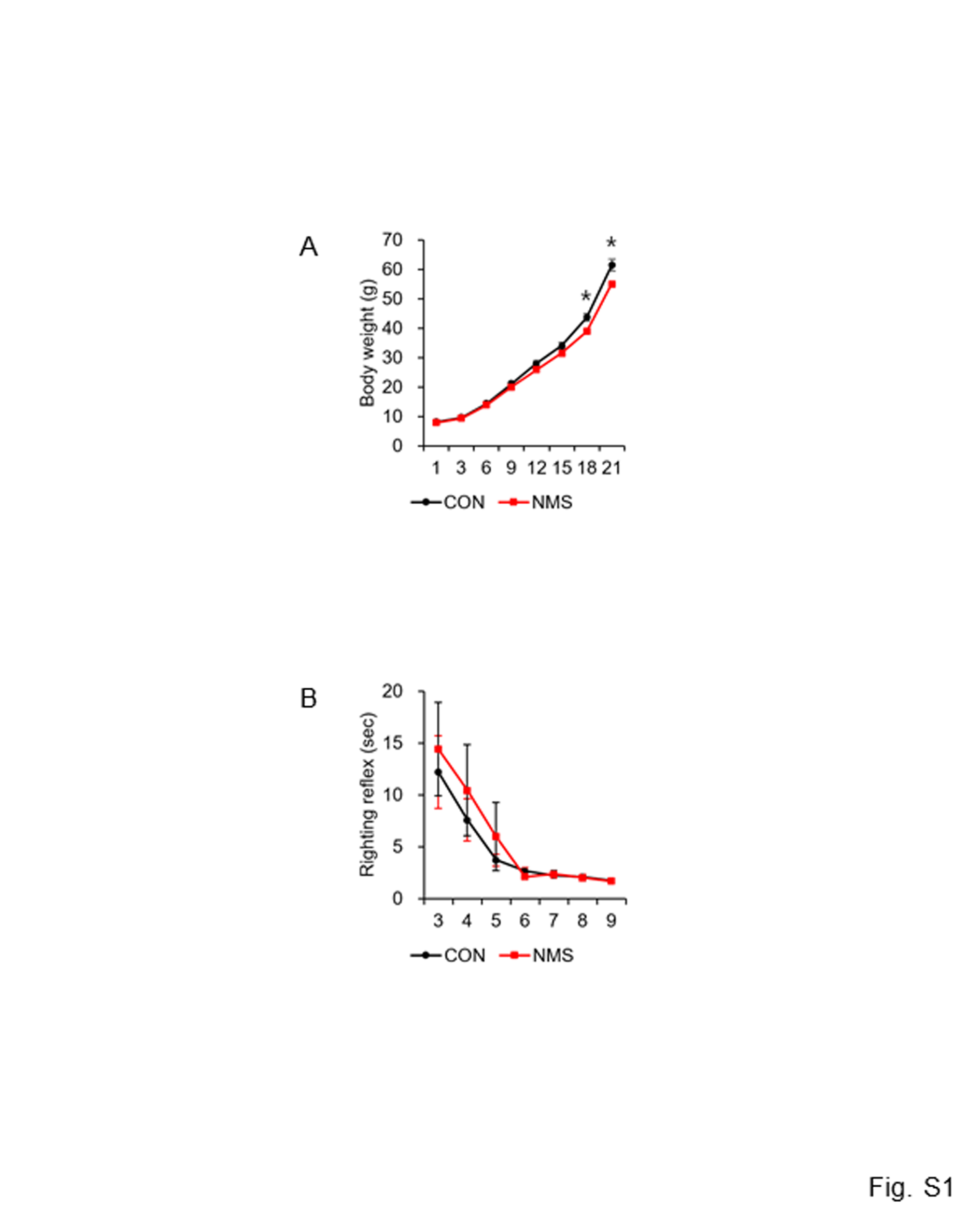

Supplement: Supplementary file 4 — Additional file 3 : Figure S1 [file 41420_2020_308_MOESM4_ESM.tif]

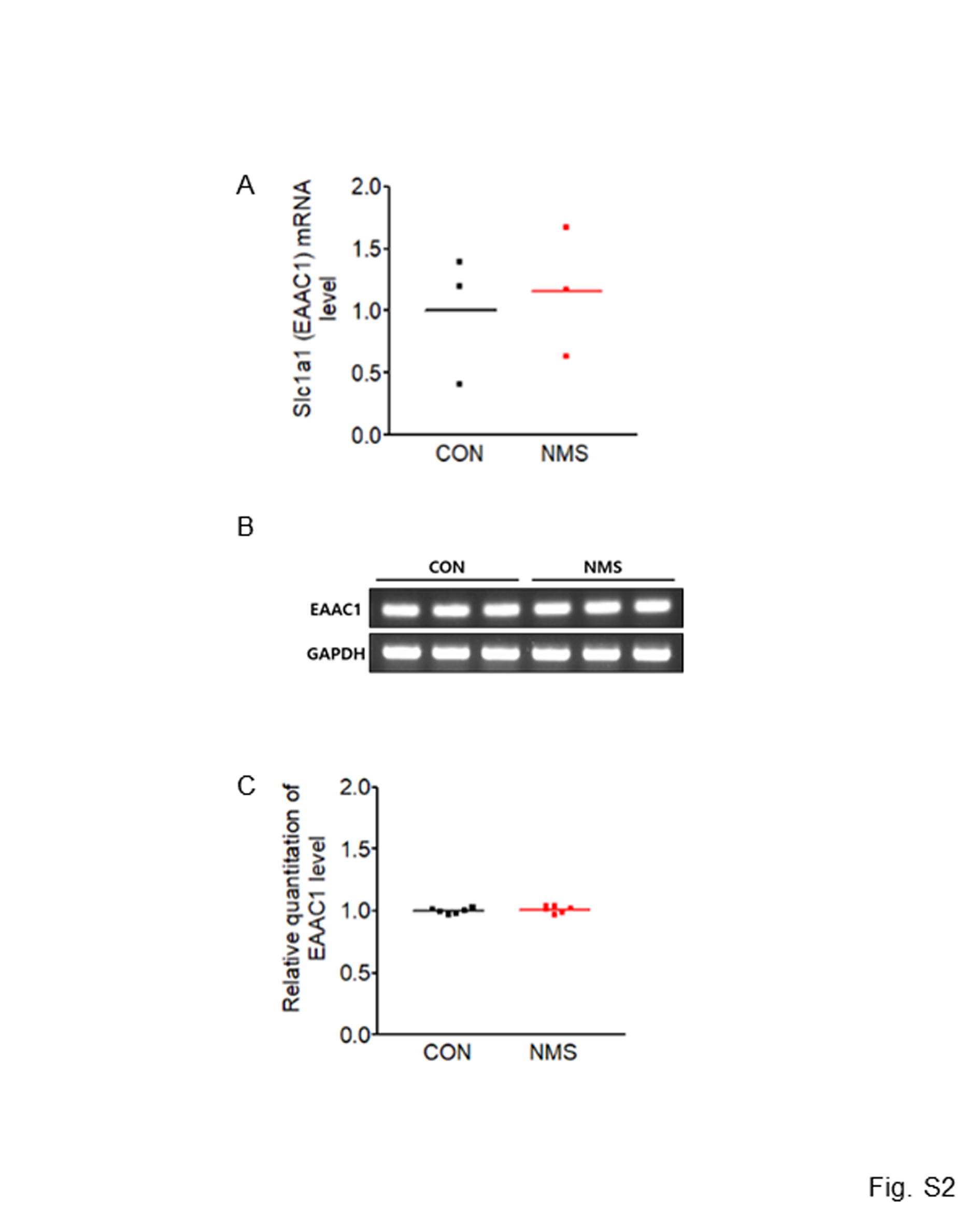

Supplement: Supplementary file 5 — Additional file 4 : Figure S2 [file 41420_2020_308_MOESM5_ESM.tif]
